# A Case of Pediatric Myopia Complicated by Vitreous Cyst: A Unique Ophthalmic Challenge

**DOI:** 10.1155/2024/4083031

**Published:** 2024-11-15

**Authors:** Abdulaziz J. Al Qattan, Abdulrahman Alasqah, Rafaa Babgi

**Affiliations:** Pediatric Ophthalmology Division, King Khaled Eye Specialist Hospital, Riyadh, Saudi Arabia

**Keywords:** congenital, myopia, pigmented cyst, visual axis, vitreous cyst

## Abstract

Vitreous cysts represent uncommon ophthalmological conditions. Most patients are asymptomatic, but a minority may experience symptoms such as floaters or blurred vision. Here, we report the case of a 2-year-old girl who was incidentally found to have a vitreous cyst in her left eye during a routine outpatient clinic visit. The cyst was observed to move with eye movements, was pigmented, lobulated, and measured 3 mm in diameter. Our patient exhibited several systemic manifestations. We recommended regular follow-up through clinical examinations and monitoring of the cyst using B-scan ultrasound.

## 1. Introduction

Intraocular cysts are considered unusual and uncommon ocular malformations. These cysts can be classified based on their location: posteriorly in the vitreous, in the retrolental space, or anteriorly in the anterior chamber [1]. They may also occur following trauma or detachment from the iris or ciliary body. Tansley first described vitreous cysts in 1899 as spheroidal bodies with pigmented lines visible on the capsule. These cysts are categorized as either primary or acquired secondary to infection, inflammation, trauma, or degeneration [2]. Typically, these cysts are discovered incidentally, though some patients may experience floaters or transient blurry vision if the cyst obscures the visual axis.

Systemic associations with vitreous cysts have not been previously reported. Our case involves a patient with tetralogy of Fallot, the most common cyanotic congenital heart disease (cCHD) that accounts for 5% of all cCHD. This condition involves aortic override and a ventricular septal defect (VSD). Narrowing of the pulmonary outflow system leads to right ventricular (RV) outflow obstruction and hypertrophy. Clinical symptoms include cyanosis and a loud murmur over the pulmonic region [3].

Ocular manifestations seen in cCHDs include retinal hemorrhages, retinal vascular tortuosity, papilledema, disc edema, uveitis, ischemic retinopathy, and central retinal vein occlusion. Retinal vascular tortuosity is the most frequently observed change [4].

## 2. Case Report

**Figure 1:**
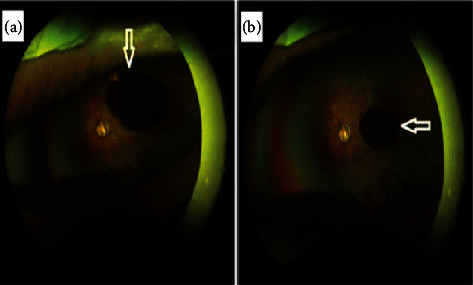
Colored fundus photo of the left eye showing pigmented free floating lobulated anterior vitreous cyst (as indicated in the arrows). (a, b) Different views of the cyst with the eye movement.

**Figure 2:**
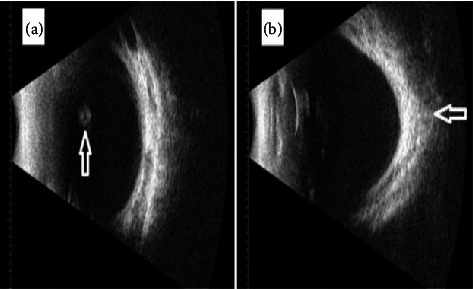
B-scan ultrasound of the left eye. (a) A floating cyst in the anterior vitreous cavity and (b) the posterior staphyloma (as highlighted in the two arrows).

A 2-year and 6-month-old girl presented to the ophthalmology clinic for a routine follow-up. She had a past medical history significant for tetralogy of Fallot, for which she underwent surgery at 6months of age. Additionally, she underwent probing and stent surgery for nasolacrimal duct obstruction in the left eye at 1 year. There was a history of consanguinity between her parents, and she is the eldest child with no siblings. On ophthalmic examination, visual acuity was fixating and following in both eyes. Teller acuity at 55 cm was 3.1 cycles/deg. There was no strabismus, nystagmus, or abnormal head posture. Cyclorefraction revealed a highly myopic fundus with −14.00 diopter in both eyes. The right eye showed a normal slit lamp examination except for a small superior sectoral cataract. The posterior segment showed a clear vitreous with a myopic fundus. In contrast, the left eye had a normal anterior segment but exhibited a myopic fundus with a pigmented, lobulated vitreous cyst measuring 3 mm in diameter (Figure 1). Ultrasound examination revealed a myopic globe with posterior staphyloma and an organized, mobile opacity detected beneath the lens (Figure 2).

Close follow-up (2 months) showed that the cyst was located in the anterior vitreous. It was single, pigmented, lobulated, floating in the anterior vitreous, and maintained the same diameter without attachment to any ocular structure. During the systemic examination, we noted a multimodal manifestation including double rows of teeth, low nasal bridge, protrusion of the chest bone, enlarged upper lip, and inability to extend or flex the pinky in both hands. Genetic testing was offered to the parents, but they declined at that time.

Given amblyopia in both eyes due to high myopia, we recommended regular follow-up with myopic correction and amblyopia treatment to monitor cyst progression and visual acuity development. Periodic examinations revealed a stable condition. Glasses and amblyopia treatment were attempted, but the patient was very uncooperative.

## 3. Discussion

Vitreous cysts can be classified into different categories based on their origin, location, and morphology. Congenital cysts originate from remnants of the hyaloid artery, which are usually grey, nonpigmented cysts, whereas acquired cysts are associated with various pathologies such as uveitis, retinal detachment, toxoplasmosis, retinoschisis, high myopia, and uveal coloboma [5]. The second classification pertains to the cyst's position, which can be within the vitreous cavity, the retrolental space, or the anterior chamber [6]. Additionally, the color of the cyst can indicate its pigmentation—brown cysts are pigmented, while yellow or gray cysts are nonpigmented [7].

These cysts can be found across a wide range of ages, typically from 5 to 68 years old, with a higher likelihood in patients aged 10–20 years [8]. However, cases have been reported in children as young as 15 months [9]. Cyst size varies from 0.15 to 12 mm in diameter and may have a lobulated, spherical, or oval shape. They can occur singly or multiply and may affect one or both eyes [5].

Awan reported that 2.7% of patients with vitreous cysts had a history of trauma in 1975 [10]. However, the correlation between cyst formation and trauma remains debatable [11]. Vitreous cysts are typically asymptomatic, but patients may complain of floaters or transient blurry vision if the visual axis is affected [12].

In our patient, the cyst exhibited characteristics consistent with literature descriptions regarding morphology, location, size, and symptoms. However, the patient's age was younger than typically reported. Other similar syndromes described in the literature include Carpenter syndrome, which presents with craniosynostosis, syndactyly, brachydactyly, and distinctive facial features such as a prominent forehead, low-set ears, and widely spaced eyes. Hypoplastic jaw has also been noted in this syndrome. Some systemic features observed in our patient, such as tetralogy of Fallot, double rows of teeth, low nasal bridge, protruding chest bone, enlarged upper lip, and inability to extend or flex the pinky in both hands, have not been previously reported. The association between vitreous cysts and high myopia has been reported in an adult patient, yet no clear pathophysiology has been described [13].

Based on ocular findings and the characteristics of the vitreous cyst in our patient, it could be a developmental cyst secondary to high myopia and posterior staphyloma.

Since our patient was asymptomatic and there was no progression of the cyst during periodic follow-up, we opted for observation. Given these systemic involvements, genetic testing should be considered. Various management modalities have been reported in the literature, including surgical removal of the cyst through pars plana vitrectomy and argon laser photocystotomy [12, 14, 15].

## 4. Conclusion

Vitreous cysts are rare ocular disorders, with most cases reported in adults and few in children. Our case is considered rare due to its systemic manifestations. Management strategies remain controversial; however, monitoring cyst progression and preserving visual potential are essential.

## Data Availability

The data supporting this study are provided by King Khaled Eye Specialist Hospital (KKESH) in Riyadh, Saudi Arabia. Due to licensing restrictions, these data are not publicly accessible. However, they may be obtained from the authors upon reasonable request and with permission from the KKESH research center.
